# FEASIBILITY AND ACCEPTABILITY OF AN EXERGAMING INTERVENTION FOR OLDER ADULTS AT RISK FOR FALLS

**DOI:** 10.1093/geroni/igz038.1609

**Published:** 2019-11-08

**Authors:** Elisa F Ogawa, Haikun Huang, Lap-Fai Yu, Philimon N Gona, Richard K Fleming, Jonathan F Bean, Suzanne G Leveille, Tongjian You

**Affiliations:** 1 New England GRECC, VA Boston Healthcare System, Boston, Massachusetts, United States; 2 University of massachusetts Boston, Boston, Massachusetts, United States; 3 George Mason University, Fairfax, Virginia, United States

## Abstract

This study examined the feasibility and acceptability of an exergaming program that utilized custom exergames, and compared it to a traditional physical exercise (control) program in older adults at risk for falls. A quasi-experimental study was conducted in older adults who lived in senior living communities. Participants enrolled in either program offered twice weekly for 8 weeks based on their residential site. Thirty-five participants enrolled in the study (mean age 77±7y) and 29 (82%) completed the follow-up assessment (exergaming: 93%; control: 73%). Overall attendance was 73% (exergaming: 79%; control: 68%), and 22 participants returned their program satisfaction form. There were no significant between-group differences in ratings of overall quality, enjoyment, instructors, peers, and facility of their assigned exercise programs. The exergaming intervention was well received and perceived as enjoyable. This study demonstrates that an 8-week exergaming intervention is feasible and acceptable for older adults at risk for falls.

